# Application of Biochar Derived From Pyrolysis of Waste Fiberboard on Tetracycline Adsorption in Aqueous Solution

**DOI:** 10.3389/fchem.2019.00943

**Published:** 2020-02-13

**Authors:** Deliang Xu, Yaxuan Gao, Zixiang Lin, Wenran Gao, Hong Zhang, Karnowo Karnowo, Xun Hu, Hongqi Sun, Syed Shatir A. Syed-Hassan, Shu Zhang

**Affiliations:** ^1^Lab of Biomass Energy and Functional Carbon Materials, College of Materials Science and Engineering, Nanjing Forestry University, Nanjing, China; ^2^School of Material Science and Engineering, University of Jinan, Jinan, China; ^3^School of Engineering, Edith Cowan University, Joondalup, WA, Australia; ^4^Faculty of Chemical Engineering, Universiti Teknologi MARA, Shah Alam, Malaysia

**Keywords:** biochar, tetracycline, fiberboard, adsorption, pyrolysis

## Abstract

In this study, biochars derived from waste fiberboard biomass were applied in tetracycline (TC) removal in aqueous solution. Biochar samples were prepared by slow pyrolysis at 300, 500, and 800°C, and were characterized by ultimate analysis, Fourier transform infrared (FTIR), scanning electron microscopy (SEM), X-ray photoelectron spectroscopy (XPS), Brunauer–Emmett–Teller (BET), etc. The effects of ionic strength (0–1.0 mol/L of NaCl), initial TC concentration (2.5–60 ppm), biochar dosage (1.5–2.5 g/L), and initial pH (2–10) were systemically determined. The results present that biochar prepared at 800°C (BC800) generally possesses the highest aromatization degree and surface area with abundant pyridinic N (N-6) and accordingly shows a better removal efficiency (68.6%) than the other two biochar samples. Adsorption isotherm data were better fitted by the Freundlich model (*R*^2^ is 0.94) than the Langmuir model (*R*^2^ is 0.85). Thermodynamic study showed that the adsorption process is endothermic and mainly physical in nature with the values of Δ*H*^0^ being 48.0 kJ/mol, Δ*S*^0^ being 157.1 J/mol/K, and Δ*G*^0^ varying from 1.02 to −2.14 kJ/mol. The graphite-like structure in biochar enables the π-π interactions with a ring structure in the TC molecule, which, together with the N-6 acting as electron donor, is the main driving force of the adsorption process.

## Introduction

Aquatic ecosystem pollution by antibiotics has received rising concerns due to their potential hazards on the aquatic biota and even human beings (Liu et al., 2012; Jang et al., 2018; Premarathna et al., 2019). The tetracycline (TC) group is one of the most widely applied antibiotics in human therapy and the livestock farming globally thanks to their comparatively low prices and efficient treatment (Liu et al., 2012; Nguyen et al., 2019). TC is hard to metabolize and was extensively excreted (up to 90%) in urine and feces from human and animals (Jang et al., 2018; Li et al., 2018; Selmi et al., 2018; Jang and Kan, 2019). It has been reported that TC was widely found in various water bodies, e.g., surface water, groundwater, and even drinking water (Jeong et al., 2010; Cao et al., 2019; Zhang et al., 2019). Antibiotics removal from water bodies is highly interesting for many researchers worldwide. Various technologies were adopted to remove TC, including biological, electrochemical, membrane, advanced oxidation process, and adsorption process (Jeong et al., 2010; Cao et al., 2019; Smyk et al., 2019). In comparison with other approaches, adsorption is outstanding for its unique advantages like easy operation, low toxicity, low energy cost, as well as high removal efficiency at low concentrations (Han et al., 2019; Regkouzas and Diamadopoulos, 2019; Shaheen et al., 2019). There are numerous effective adsorbents such as activated carbon (Xiang et al., 2019), carbon nanotubes (Xiang et al., 2019), zeolite (Wang et al., 2019), chitosan (Ahamad et al., 2019), etc. However, those adsorbents are relatively unaffordable when dealing with large scale of wastewater. Therefore, alternative low-cost adsorbents are highly demanded for TC removal. Biochar, a by-product of biomass pyrolysis, has been attracting huge attention as a promising cost-effective adsorbent due to its properties of abundant functional groups, porous structure, rich aromatic structures, and environmental friendly nature (Ahmad et al., 2014; Patra et al., 2017; Maleki et al., 2018).

On the other hand, fiberboard as a major wood composite possesses a huge gross production capacity (~55.54 million m^3^ in 2013 in China), which leads to a large quantity of waste fiberboard (Gan et al., 2004; Wu et al., 2012; Liu et al., 2014). Nowadays, waste fiberboard is generally disposed of by burning, which is uneconomical and extremely harmful to environment as well as human health (Liu et al., 2014; Zhang et al., 2014). As a result, dealing with such amount of waste fiberboard is of critical importance for both economic and environmental concerns. One effective and economical method to better utilize those waste fiberboard is to prepare biochar via pyrolysis. Previous studies have proved that biochar derived from waste fiberboard biomass is a promising adsorbent for removal of heavy metal and organics, with or even without further modification (Wu et al., 2012, 2014; Liu et al., 2014; Pan et al., 2016, 2018). It has been reported that biochar from fiberboard biomass usually possesses high content of N-containing groups, which is beneficial to the adsorption process due to the enhanced alkaline property and wettability (Wu et al., 2012, 2014; Zhang et al., 2014; Zhan et al., 2019). This also addresses the issue of high cost adsorbents along with TC removal.

Therefore, this study aims to prepare and characterize biochar derived from pyrolysis of waste fiberboard, which was then utilized for TC removal in aqueous. The properties of TC solutions (i.e., ionic strength, initial concentration, and pH) were varied to examine the changes in adsorption behavior and capacities by the waste-derived chars, while the adsorption isotherms and thermodynamics analysis were also conducted to explore the fundamental mechanism. Overall, this study has made appreciable progress for the understanding TC adsorption on biochar derived from waste fiberboard.

## Materials and Methods

### Materials

TC was purchased from Macklin Biochemical (Shanghai, China). HCl was sourced from Nanjing Chemical Reagent (Nanjing, China) while both NaOH and NaCl were supplied by Sinopharm Chemical Reagent (Shanghai, China). The above chemical reagents are all of analytical grades. All solutions used in this study were prepared with deionized water.

### Preparation and Characterization of Biochar

The fiberboard biomass samples used in this study were sourced from Dare Wood-Based Panels Group (Jiangsu, China). Then, the biomass samples were cut, ground, and sieved to a size of 74–200 μm. The biochar samples were produced by slow pyrolysis of fiberboard biomass using a laboratory-scale tube furnace (YGDL-1200, Shanghai yuzhi electromechanical equipment Co., Ltd, China) at 300, 500, and 800°C, respectively. All pyrolysis experiments were performed at a heating rate of 10°C/min, a holding time of 1 h, and a N_2_ flow rate of 0.5 L/min. Hereafter, the biochar samples are referred to as BCXXX, where the prefix “BC” denotes biochar while the suffix “XXX” represents the pyrolysis temperature (in degrees Celsius).

The BC samples were subjected to the following characterizations. The elemental analysis of biochar was performed via an elemental analyzer (Perkin-Elmer 2400 Series II, USA). Scanning electron microscopy (SEM; JSM-7600F, Japan Electronics, Japan) and X-ray diffraction (XRD; Ultima IV, Rigaku, Japan) were adopted to observe the surface morphology and the crystalline structures of biochar samples. The functional groups of biochar were studied via FTIR spectrometry (Vertex 80V, Bruker, Germany). The surface elemental composition of biochar was determined by XPS (AXIS Ultra DLD, Shimadzu, UK). BET surface area and pore size distribution of the biochar samples were measured on an autosorb specific surface area analyzer (Quantachrome, USA) with N_2_ as the adsorbate at 77 K.

The pH of zero point charges (pH_PZC_) of selected biochar samples were recorded based on a method detailed elsewhere (Liu et al., 2011; Jang et al., 2018). In brief, 50 ml of NaCl solutions (0.01 M, to maintain the ion strength of the solution) with pH ranging from 2 to 10 (adjusted by adding appropriate amount of 0.5 M HCl or NaOH solution) was placed in 250-ml conical flasks. Then 0.1 g of biochar was added to the solution followed by purging N_2_ gas to eliminate the effect of CO_2_. The final pH of solution was recorded after stirring for 48 h at ~25°C in a sealed flask. Finally, the pH_pzc_ was calculated based on the ΔpH = 0, where ΔpH is equal to the final pH minus the initial pH. All experiments were performed at least twice.

### Batch Adsorption Experiments

A stock TC solution of 500 ppm was prepared by dissolving 0.05 g of TC together with 0.025 g of NaOH (to increase its solubility) in 100 ml of water. Then, the stock solution was diluted to desired concentrations with pH adjusted to the desired value for adsorption experiments. It should be noted that all TC solutions contain 0.1 M of NaCl to maintain the ionic strength, if not specified. For the adsorption experiments, 0.25 g of biochar sample was added into 100 ml of TC solution (20 ppm, pH = 7) in a 250-ml conical flask and stirred for 96 h at ~25°C. At certain time intervals, ~5 ml of solution was withdrawn and filtered by a 0.22-μm Millipore filter. The concentrations of TC were determined by a UV-Vis spectrophotometer (WFZ UV-2000, Unico, USA) at 360 nm. It should be noted that to better determine the concentration of TC solution, different standard curves at different pH values (i.e., 2–10) were adopted. The pH of the filtrates was also recorded. Since the adsorption equilibrium is achieved after 48 h, all following adsorption experiments were conducted for 48 h. For studying the influence of ionic strength, the concentration of NaCl was adjusted from 0.0 to 1.0 M. To study the effect of biochar dosage, the biochar dose ranged from 1.5 to 3.5 g/L. When examining the role of pH values, the initial pH of TC solution was set from 2 to 10. The initial TC concentration was varied from 2.5 to 60 ppm for obtaining the adsorption equilibrium isotherm. The removal efficiency was calculated based on the following formula:

(1) $$\begin{array}{l} R=\frac{C_{0}-C_{e}}{C_{0}}\times 100\% \end{array}$$

where *R* is removal efficiency and *C*_0_ and *C*_e_ are the initial and equilibrium concentration of TC (mg/L).

The adsorption capacity *q*_e_ was calculated according to the following formula:

(2) $$\begin{array}{l} q_{e}=\frac{C_{0}-C_{e}}{m}V \end{array}$$

where *C*_0_ and *C*_e_ are the initial and equilibrium concentration of TC (mg/L), *V* is the volume of TC solution (L), *m* is the weight of biochar (g), and *Q*_e_ is the adsorption capacity (mg/g).

The Langmuir and Freundlich isotherm modes were adopted to evaluate the reaction behavior between TC and biochar, which can be expressed by the following equations:

(3) $$\begin{array}{l} q_{e}=\frac{{q_{\text{max}}k}_{L}C_{e}}{1+k_{L}C_{e}} \end{array}$$

(4) $$\begin{array}{l} q_{e}=k_{F}{c_{e}}^{\frac{1}{n}} \end{array}$$

where *C*_e_ is equilibrium concentration of TC (mg/L), *q*_e_ is the adsorption capacity (mg/g), *q*_max_ is the maximum adsorption capacity (mg/g), *K*_L_ (L/mmol) and *K*_F_ [(mmol/g)·(L/mmol)^1/n^] are Langmuir and Freundlich constants, respectively, and *n* is another Freundlich constant that is related to adsorption intensity.

A thermodynamic study was completed by using temperatures of 25, 35, and 45°C. Equations (5)–(7) were applied to determine the change in the standard Gibbs free energy (Δ*G*^0^), enthalpy (Δ*H*^0^), and entropy (Δ*S*^0^), respectively:

(5) $$\begin{array}{l} K_{c}=\frac{q_{e}}{C_{e}} \end{array}$$

(6) $$\begin{array}{l} \Delta G^{0}=-RTlnK_{c} \end{array}$$

(7) $$\begin{array}{l} \Delta G^{0}=\Delta H^{0}-T\Delta S^{0} \end{array}$$

where *K*_C_ (dimensionless) means the apparent equilibrium constant; *R* is the gas constant, which is 8.314 J/mol/K; and *T* is the absolute temperature (K). When plotting lnK_C_ against 1/T, a straight line can be found with Δ*H*^0^ and Δ*S*^0^ being the intercept and slope, respectively. All experiments were again repeated at least twice.

## Results and Discussion

### Characteristics of Biochar

Table 1 shows that the ash content of biochar samples gradually increases from 5.8% of BC300 to 7.9% of BC800. The higher inorganic content for BC800 is due to the enhanced decomposition of organic components in fiberboard biomass at a higher pyrolysis temperature. The elemental compositions of biochar samples derived from fiberboard biomass are also shown in Table 1. As can be seen, the C content (83.5–87.6%) in biochar samples prepared at different temperatures are relatively high, while both the H (0.9–3.0%) and O content (8.0–10.0%) are very low. It should be noted that the N contents of biochar samples prepared from fiberboard biomass in this study are 3.2–3.5%, which are apparently higher than biochar samples derived from other biomass feedstock. This can be attributed to the presence of glue containing urea in fiberboard. Another phenomenon is that with increasing pyrolysis temperature, C content slightly rises while both H and O contents decrease. This indicates a higher carbonization degree of biochar with less hydrophilic surfaces (Wang et al., 2017). As a result, the H/C reduces from 3.6 to 1.1 and the O/C gradually reduces from 12.0 to 9.2, indicating a higher aromatization degree and the graphite-like structure of higher temperature biochar (Selmi et al., 2018; de Jesus et al., 2019).

**Table 1:** Proximate and elemental analysis of biochar samples.

**Samples**	**Proximate analysis (wt.%, ar^a^)**				**Elemental analysis (wt.%, daf^b^)**					
	**Moisture**	**Volatile matter**	**Fixed carbon**	**Ash**	**C**	**H**	**O^c^**	**N**	**H/C^d^**	**O/C^d^**
BC300^e^	4.8	42.8	46.6	5.8	83.49	2.98	9.99	3.54	3.6	12.0
BC500^e^	6.4	24.7	62.2	6.7	85.57	1.56	9.71	3.16	1.8	11.3
BC800^e^	8.7	17.7	65.7	7.9	87.61	0.95	8.02	3.42	1.1	9.2

a *As received basis*.

b *Dry and ash-free basis*.

c *By difference*.

d *Atomic ratio*.

e *BCXXX stands for biochar prepared at XXX°C; XXX can be 300, 500, and 800*.

**Figure 1:**
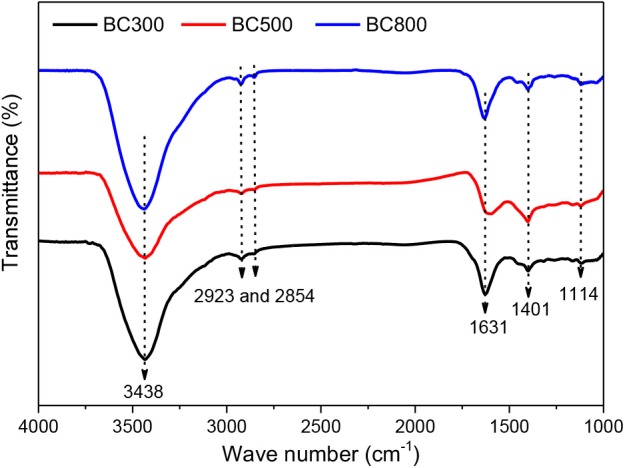
FTIR spectra of biochar samples prepared at 300, 500, and 800°C. BCXXX stands for biochar prepared at XXX°C; XXX can be 300, 500, and 800.

FTIR spectra in Figure 1 also prove the existence of a graphite-like structure as well as oxygen-content functional groups. The peaks at wavenumbers of 1,114 and 1,400 cm^−1^ are attributed to alcoholic C–O and C–N stretching, respectively (Pan et al., 2018). The strong peak at 1,630 cm^−1^ is assigned to ether the aromatic stretching of C–C groups or the stretching of C=O, while the peaks at wavenumbers 2,923 and 2,854 cm^−1^ are due to the stretching vibrations of C–H groups (Liu et al., 2012; Ahsan et al., 2018; Pan et al., 2018). The broad band at 3,438 cm^−1^ could be assigned to the overlapping of –OH and –NH stretching (Pan et al., 2018; de Jesus et al., 2019).

To further probe the surface elemental composition and the contents of both O-containing and N-containing functional groups, XPS analysis on biochar samples were carried out (see Table 2 and Figure S2). The surface C content increases from 76.8% of BC300 to 86.4% of BC800, while the surface O content decreases from 20.4 to 11.3%. These results suggest a higher aromatization degree under higher pyrolysis temperature, which is consistent with elemental analysis aforementioned. It is obvious that the total content of O-containing functional groups (i.e., C–O and C=O groups) becomes lower from 35.5 to 19.4%. According to previous studies (Liu et al., 2011; Wang et al., 2017; Jang et al., 2018; de Jesus et al., 2019), both the graphite-like structure and the O-containing functional groups contribute to the adsorption performance of biochar. Moreover, there are four types of N-containing functional groups: pyridinic nitrogen (N-6), pyrollic nitrogen (N-5), quaternary nitrogen (N-Q), and oxidized nitrogen (N-O). All N-containing groups are located at the edges of the graphene structure, except N-Q (Wu et al., 2013). It can be seen in Table 2 that the dominant type is N-5 followed by N-6 and N-Q in sequence. As reported, N-containing groups may contribute to the adsorption performance of biochar (Wu et al., 2012, 2013, 2014; Zhang et al., 2014; Zhan et al., 2019).

**Table 2:** Elemental composition, oxygen-containing functional groups, and nitrogen-containing functional groups of biochar surface from XPS analysis.

**Samples**	**Elemental composition** **(At.%)**			**Oxygen-containing functional group** **(At.%)**			**Nitrogen-containing functional group** **(At.%)**			
	**C**	**N**	**O**	**C–C (284.6 eV)**	**C–O** **(286.2 eV)**	**C=O (287.6 and 289.1 eV)**	**N-6** **(398.5 eV)**	**N-5** **(400.3 eV)**	**N-Q** **(401.2 eV)**	**N-X** **(403.8 eV)**
BC300^a^	81.2	2.5	16.2	64.5	32.4	3.1	21.9	48.2	22.5	7.4
BC500^a^	86.5	2.0	11.5	73.1	25.9	3.0	31.9	47.8	14.5	5.8
BC800^a^	89.2	2.0	8.8	80.6	16.7	2.7	35.7	35.1	22.4	6.8

a *BCXXX stands for biochar prepared at XXX°C; XXX can be 300, 500, and 800*.

The surface morphology of biochar samples is presented in Figure S1 and the surface area is listed in Table 3. As can be observed, the surfaces of biochar samples are not very rough with its surface areas being very low (32.2 and 34.9 m^2^/g for BC300 and BC500, respectively). BC800 seems to show more coarse surface texture than others, which is consistent with the results of BET analysis. The surface area of BC800 significantly improves to 135.1 m^2^/g due to the server decomposition of organic components in biomass feedstock at elevated temperature (Lian et al., 2014). A similar trend is also found for the pore volume, and both trends remain consistent with previous reports (Aller, 2016; Wang et al., 2017). The higher surface area and pore volume are particularly favorable when using biochar as adsorbents (de Jesus et al., 2019). Taking all above characterizations into consideration, biochars prepared from fiberboard biomass could be potential adsorbents, especially BC800.

**Table 3:** Pore structure of biochar samples.

**Samples**	**Surface area (m^**2**^/g)**	**Pore volume (cm^**3**^/g)**	**Average pore size (nm)**
BC300^a^	32.2	0.022	2.5
BC500^a^	34.9	0.033	4.3
BC800^a^	135.1	0.108	3.3

a *BCXXX stands for biochar prepared at XXX°C; XXX can be 300, 500, and 800*.

### TC Adsorption by Biochar

#### Adsorption Ability of BC300, BC500, and BC800

**Figure 2:**
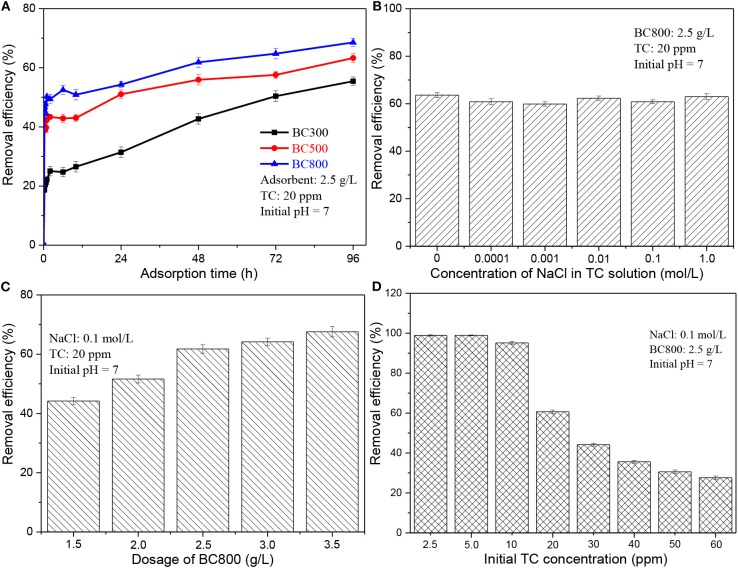
Adsorption performance of **(A)** BC300, BC500, and BC800 on TC; **(B)** BC800 on TC with different concentrations of NaCl; **(C)** BC800 on TC with different biochar dosage; **(D)** BC800 on TC with different initial concentration. BCXXX stands for biochar prepared at XXX°C; XXX can be 300, 500, and 800.

**Figure 3:**
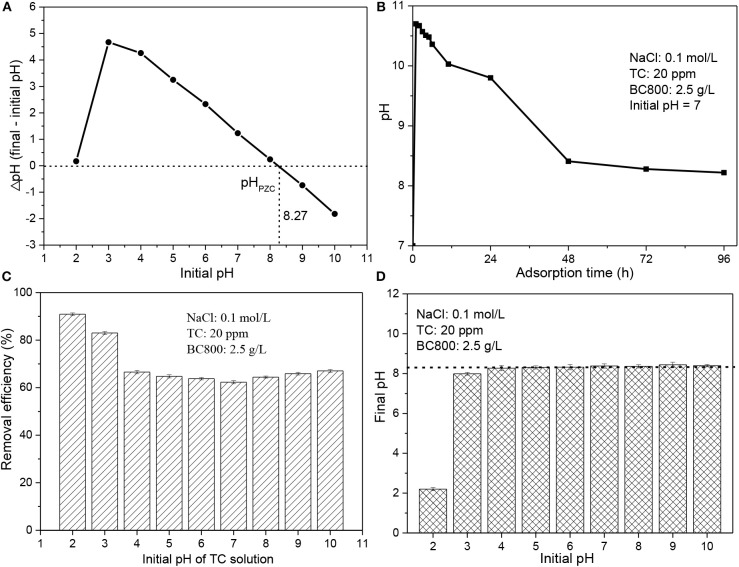
**(A)** pH_PZC_ of BC800; **(B)** the pH of TC solution with adsorption time; **(C)** adsorption performance of BC800 on TC solution with different initial pH; and **(D)** the final pH of TC solution after adsorption benchmarking against its initial pH. pH_PZC_ stands for the pH of zero point charges; BC800 stands for biochar prepared at 800°C.

Figure 2A presents the adsorption capacity of biochar samples prepared at different pyrolysis temperatures on TC adsorption. It clearly shows that biochar prepared at a higher pyrolysis temperature performs a better adsorption capacity on TC. As aforementioned (see section Characteristics of Biochar), BC800 has a higher aromatization degree and a bigger surface area, compared to BC300 and BC500. A previous study has also proved a positive influence of the surface area of biochar on its adsorption capacity (Wang et al., 2017). However, the differences in adsorption capacity among three biochars are vastly different from the difference in their surface areas, implying that the measured BET surface area may not be the main factor in determining the adsorbing ability. Based on the properties of biochar, possible adsorption mechanisms include pore-filling, hydrogen bonds, hydrophobic effect, electrostatic interactions, and π-π interactions (Wang et al., 2017; de Jesus et al., 2019). It has been reported that π-π interactions are one of the major mechanisms governing TC adsorption by biochar (Wang et al., 2017). Biochar serves as a π-electron donor, which could be attributed to its graphite-like structure while TC acts a π-electron acceptor due to its aromatic ring structure (Wang et al., 2017; de Jesus et al., 2019). A higher pyrolysis leads to a higher degree of graphitization of biochar samples. The crystallinity of obtained biochar samples was characterized by XRD analysis, as is shown in Figure S3. Three biochar samples exhibit a broad diffraction peak at 23.5° with the intensity of BC800 being the highest, which is attributed to the (002) crystal plain of graphitic structure. BC800 also exhibit a board diffraction peak at 44.3° with low intensity, which is assigned to the (100) crystal plain of graphitic structure. XRD analysis confirms the high graphitization degree of BC800. Furthermore, the abundant N-6 in BC800 (see Table 2) also contributes to its high absorbing ability as N-6 possesses an unshared pair of electrons, thus enhancing its performance as electron donor when interacting with TC. It has been reported that the adsorption energy of morpholine with N-6 is much higher than those with other types of N-containing functional groups (e.g., N-5, Q-N, etc.) (Li et al., 2019). Therefore, BC800 shows the best adsorption capacity with removal efficiency being 68.6%. It is also reported that O-containing functional groups can act as hydrogen-bond acceptors, thus increasing the adsorption capacity. However, in this study, BC300 possesses the highest content of O-containing functional groups while having the lowest adsorption capacity. This indicates that the hydrogen bond interactions do not dominate the adsorption behavior. Hereafter, only BC800 is chosen to use as adsorbent. It is also worth noting that when the adsorption time is longer than 48 h, the increase of removal efficiency becomes extremely slow, which means that the adsorption equilibrium is approached after 48 h. The final pH of the solution also remains stable after 48 h (see Figure 3B). Hence, an adsorption time of 48 h is adopted for the following studies.

#### Effect of Ionic Strength

The real wastewater system is complex and often contains salts besides organic pollutants (Liu et al., 2019). Besides, in this study, NaOH and HCl are used to adjust the pH of TC solution, so the TC solution contains NaCl. The existence of salts may influence the removal of pollutants. Therefore, it is necessary to investigate the effect of ionic strength, and the results are shown in Figure 2B. It is obvious that the removal efficiency is similar (59.9–63.6%) with the concentration of NaCl ranging from 0 to 1.0 mol/L. This suggests that the existence of salts does not obviously affect the removal process. To further eliminate the influence of ionic strength, 0.1 mol/L of NaCl is added to TC solution for the following studies.

#### Effect of Biochar Dosage

Figure 2C presents that increasing biochar dosage from 1.5 to 2.5 g/L greatly improves removal efficiency from 44.2 to 62.3%, while further increasing biochar dosage to 3.5 g/L only slightly improves removal efficiency to 67.6%. This improvement can be attributed to the more adsorption sites from the enlarged surface area of adsorbent at a larger dosage level (Ahsan et al., 2018; Alidadi et al., 2018). The slow increase of removal efficiency after 2.5 g/L may be due to the agglomeration of biochar particles that reduces the total effective surface area and thereby reduces total sorption sites (Ahsan et al., 2018; Alidadi et al., 2018). As a result, 2.5 g/L of BC800 dosage is employed in this study.

#### Effect of Initial TC Concentration

A negative correlation between TC removal efficiency and its initial concentration is observed in Figure 2D. When the TC initial concentration is lower than 10 ppm, more than 95% TC is removed. Increasing TC initial concentration to 20 ppm leads to a significant reduction of removal efficiency to ~60%. The removal efficiency slowly reduced from 44.2 to 27.6% with TC initial concentration growing from 30 to 60 ppm. This could be attributed to the restriction of the adsorption process at a high antibiotic loading level caused by the limited number of effective adsorption sites (Marzbali et al., 2016; Ahsan et al., 2018; Alidadi et al., 2018). The alleviated negative effect of initial TC concentration on the removal efficiency at high concentrations (20–60 ppm) may come from the increased TC concentration gradients between liquid phase and solid surfaces (Ahsan et al., 2018; Alidadi et al., 2018). An initial TC concentration of 20 ppm is used in this study.

#### Effect of Initial pH

Solution pH is also an important parameter to consider for the effective adsorption process, which affects the properties of both adsorbents and pollutants (Liu et al., 2012; Marzbali et al., 2016; Ahsan et al., 2018; Alidadi et al., 2018; Jang et al., 2018; Selmi et al., 2018; Jang and Kan, 2019; Nguyen et al., 2019; Premarathna et al., 2019). The pH_PZC_ of BC800 was 8.27 (see Figure 3A), which is relatively high. This may be due to the presence of N-containing groups that lead to BC800 being more alkaline (Wu et al., 2012). The surface of BC800 is negatively charged at pH lower than 8.27 while positively charged at pH higher than 8.27. Based on the pKa values studied previously (Marzbali et al., 2016; Selmi et al., 2018), TC molecules are positively charged when pH < 3.3, neutrally charged when pH ranges from 3.3 to 7.8, and negatively charged when pH > 7.8. It should be noted that the pH value dramatically rises to 10.7 within 10 min and then significantly reduces to ~8.35, which is similar with the pH_PZC_ (8.27) after 48 h and finally stable at this pH value (see Figure 3B). A similar trend was also found when the initial pH is 3–10. When the initial pH is 2, its final pH slightly increases to 2.2, as can be seen in Figure 3D. This suggests that the biochar samples serve as a buffer that releases some acid matters or alkali matters to react with NaOH or HCl. Consequently, both the TC molecules and biochar particles are charged ether positively (initial pH is 2) or negatively (initial pH is 3–10). It has been reported that if both the adsorbent and the adsorbate are negatively or positively charged, the adsorption process will be repelled due to the electrostatic repulsion (Li et al., 2018). However, in this case, the maximum removal efficiency (90.9%) was achieved at a pH value of 2, as can be seen in Figure 3C. The removal efficiency dramatically decreases to 83.0% (pH = 3) and then gradually reduces to 62.3% with pH increasing to 7, followed by a gradually increase to 67.1% with pH increasing. The results indicate that electrostatic attraction does not play an important role in the TC adsorption. Similar phenomenon also found in other studies and other mechanisms should be mainly responsible for TC adsorption, such as graphene-like structure in adsorbents (Li et al., 2018). Overall, natural environmental pH is appropriate for TC adsorption by BC800.

### Adsorption Isotherms and Thermodynamics Analysis

**Figure 4:**
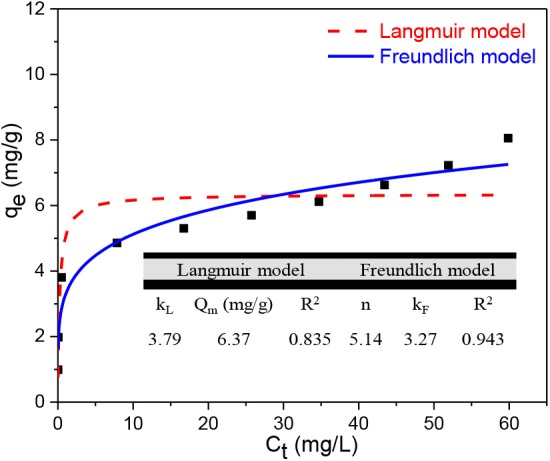
Sorption isotherms of TC using BC800 (*T* = 298 K).

Both the Langmuir and Freundlich models were adopted to study the adsorption isotherms with results shown in Figure 4. As can be seen, the *R*^2^ value of the Langmuir model is 0.85 and is lower than that of the Freundlich model, which is 0.94. This suggests that both models are suitable to predict the experimental data although TC adsorption is better explained by the Freundlich model. The Freundlich model describes both physical and chemical adsorption onto heterogeneous surfaces (McKay et al., 1982; Alidadi et al., 2018). The Freundlich consistent *n* is 5.14, which means adsorption is favorable and BC800 is not so heterogeneous on its surface (Alidadi et al., 2018). A *K*_F_ value of 3.27, which is related to adsorption capacity, also supports the adsorption process. The Langmuir model explains monolayer adsorption onto homogeneous surfaces (Doltabadi et al., 2016; Alidadi et al., 2018). A dimensionless equilibrium parameter *R*_L_ can explain the nature of the Langmuir model, which is favorable (0 < *R*_L_ < 1), unfavorable (*R*_L_ > 1), irreversible (*R*_L_ = 0), or linear (*R*_L_ = 1), which can be calculated using the following equation:

(8) $$\begin{array}{l} R_{L}=\frac{1}{1+K_{L}C_{0}} \end{array}$$

The *K*_L_ value is 3.79, which makes the *R*_L_ 0.16. This indicates that BC800 is in favor of TC adsorption with maximum adsorption capacity being 6.37 mg/g. Taking the above considerations together, it can be concluded that the surfaces of BC800 are neither very heterogeneous to follow Freundlich model nor entirely homogenous to follow Langmuir model.

**Figure 5:**
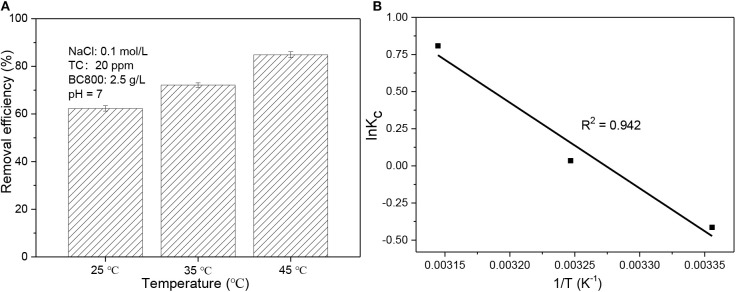
**(A)** Adsorption performance of BC800 on TC solution at 25, 35, and 45°C; **(B)** 1/T vs. lnK_C_ plot. BC800 stands for biochar prepared at 800°C.

As is known to all, temperature significantly affect the adsorption process. TC adsorption was determined at 25°C (298 K), 35°C (308 K), and 45°C (318 K) and the results are presented in Figure 5. It is obvious that with adsorption temperature rising, the removal efficiency improves greatly from 62.3% at 25°C to 84.9% at 45°C. This implies TC adsorption process is endothermic which agrees with other reports (Wang et al., 2017; Ahsan et al., 2018; Selmi et al., 2018). It is also proved by a positive Δ*H*^0^ value which is 48.0 kJ/mol (see Table 4). This result also indicates that a higher temperature is more satisfactory for adsorption process, which may be attributed to the improvement in diffusion rate of TC (Wang et al., 2017). Similar to Δ*H*^0^, Δ*S*^0^ is also positive with its value being 157.1 J/mol/K. This reveals that randomness at the TC-biochar interface is higher compared to concentrated aqueous phase (Wang et al., 2017; Selmi et al., 2018). As to Δ*G*^0^, its value is positive but very low at 298 K (1.02 kJ/mol) and then turns negative with the magnitude of Δ*G*^0^ rising with sorption temperature becoming higher. This again suggests that the sorption process is more thermodynamically preferable at a higher temperature. Basically, the Δ*G*^0^ value of physical sorption is in the range of 0–20 kJ/mol. Therefore, the adsorption process in this study is mostly physical in nature with the value of Δ*G*^0^ varying from 1.02 to −2.14 kJ/mol.

**Table 4:** Thermodynamic parameters for the adsorption of TC on BC800 at different temperatures.

***T* (K)**	***K*_**C**_**	**Δ*G*^**0**^ (kJ/mol)**	**Δ*H*^**0**^ (kJ/mol)**	**Δ*S*^**0**^ (J/mol/K)**
298	0.66	1.02	48.0	157.1
308	1.04	−0.09		
318	2.24	−2.14		

### Further Discussion

Compared with other biochar samples, the adsorption capacity of BC800 on TC in this study is not very advantageous (see Table S1). For example, Wang et al. could achieve a TC adsorption capacity of 13.85 mg/g using rice straw-derived biochar, while the similar performance of biochar prepared from the sewage sludge was also reported by Yang et al. At similar pyrolysis temperatures, agricultural residue (e.g., rice straw) could be decomposed more significantly than wood-based materials, thus leading to a better porous structure. One of the disadvantages of using straws as precursor for preparing functional carbon materials is the high content of inorganics, which will cause difficulty in further modification (e.g., carbonization and/or graphitization) and also may lead to a secondary pollution to environment. The high adsorption capacity from the sludge is due to the activation process by the ferric compounds. Comparatively, this study uses waste fiberboard to prepare biochar adsorbent containing limited inorganic metals with potentials to be further modified and upgraded by simple methods. Additionally, the fiberboard featuring high contents of heteroatoms (e.g., N and O) may endow the resulting biochars with special characteristics when being further carbonized. Therefore, this work is a preliminary study on exploring the effectiveness of fiberboard-derived biochars as adsorbents for organic pollutants. Subsequently, more investigation by fine modifications would be necessary to examine its potentials as environmental remediating green materials in the near future.

**Figure 6:**
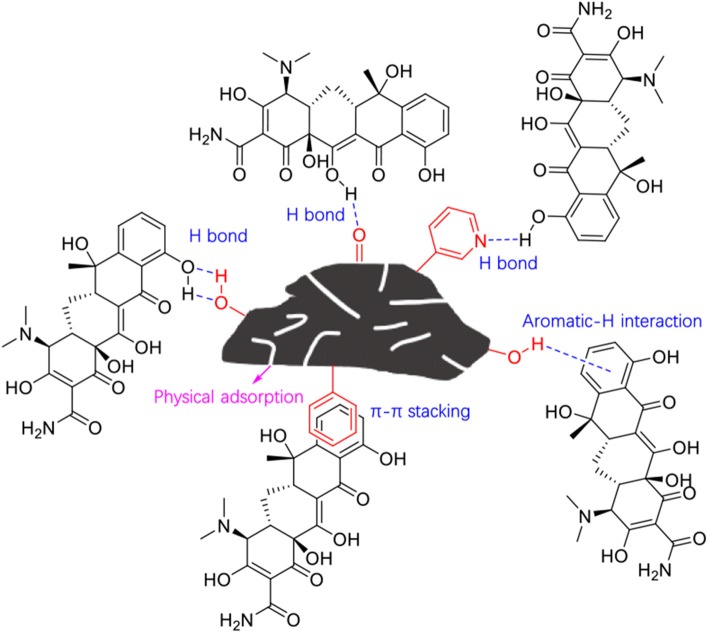
Proposed mechanisms of the adsorption process.

As discussed above, the adsorption performance of BC800 was mainly attributed to its relatively high surface area, the π-π interactions with TC, as well as the high content of N-6 as electron donor. The adsorption mechanism could thus be proposed as shown in Figure 6. This study has clearly indicated the feasibility of volarizing waste fiberboard into valuable products and the important role of inherent heteroatoms in the biomass.

## Conclusions

This study shows that the biochars derived from waste fiberboard biomass can be used as adsorbent for TC removal, especially BC800 with removal efficiency being 68.6%. This is attributed to its higher aromatization degree and a bigger surface area. The π-π interactions between the graphite-like structure of biochar and the ring structure of TC dominate the adsorption mechanism. The high content of N-containing groups (especially N-6) also contributes to the adsorption performance of biochar. The ionic strength plays an insignificant role in the adsorption process, while both the biochar dosage and the initial TC concentration significantly affect the removal efficiency. The maximum adsorption capacity is obtained at a pH of 2. The results suggest that electrostatic attraction has limited influence on the adsorption process and natural environmental pH is appropriate for TC adsorption. An isotherm study indicates that the Freundlich model fits better than the Langmuir model. Thermodynamic analysis shows that both the value of Δ*H*^0^ and Δ*S*^0^ are positive, which suggests that the adsorption process of TC on biochar is thermodynamically favorable. The adsorption process is mostly physical adsorption due to the very low values of Δ*G*^0^.

## Data Availability Statement

All datasets generated for this study are included in the article/Supplementary Material.

## Author Contributions

SZ, WG, and DX: conceptualization. SZ and WG: methodology and funding acquisition. YG and DX: validation. WG and DX: formal analysis. YG, KK, and ZL: investigation. SZ: resources and project administration. WG and HZ: data curation. DX and WG: writing—original draft preparation and visualization. XH, HS, KK, and SS-H: writing—review and editing. WG and SZ: supervision.

### Conflict of Interest

The authors declare that the research was conducted in the absence of any commercial or financial relationships that could be construed as a potential conflict of interest.

## References

[B1] Ahamad T. Ruksana Chaudhary A. A. Naushad M. Alshehri S. M. (2019). Fabrication of MnFe_2_O_4_ nanoparticles embedded chitosan-diphenylureaformaldehyde resin for the removal of tetracycline from aqueous solution. Int. J. Biol. Macromol. 134, 180–188. 10.1016/j.ijbiomac.2019.04.204 31075335

[B2] Ahmad M. Rajapaksha A. U. Lim J. E. Zhang M. Bolan N. Mohan D.. (2014). Biochar as a sorbent for contaminant management in soil and water: a review. Chemosphere 99, 19–33. 10.1016/j.chemosphere.2013.10.071 24289982

[B3] Ahsan M. A. Islam M. T. Hernandez C. Castro E. Katla S. K. Kim H. (2018). Biomass conversion of saw dust to a functionalized carbonaceous materials for the removal of Tetracycline, Sulfamethoxazole and Bisphenol A from water. J. Environ. Chem. Eng. 6, 4329–4338. 10.1016/j.jece.2018.06.040

[B4] Alidadi H. Dolatabadi M. Davoudi M. Barjasteh-Askari F. Jamali-Behnam F. Hosseinzadeh A. (2018). Enhanced removal of tetracycline using modified sawdust: Optimization, isotherm, kinetics, and regeneration studies. Process Safety Environ. Protect. 117, 51–60. 10.1016/j.psep.2018.04.007

[B5] Aller M. F. (2016). Biochar properties: transport, fate, and impact. Crit. Rev. Environ. Sci. Technol. 46, 1183–1296. 10.1080/10643389.2016.1212368

[B6] Cao J. Lai L. Lai B. Yao G. Chen X. Song L. (2019). Degradation of tetracycline by peroxymonosulfate activated with zero-valent iron: performance, intermediates, toxicity and mechanism. Chem. Eng. J. 364, 45–56. 10.1016/j.cej.2019.01.113

[B7] de Jesus J. H. F. da S. Matos T. T. da C. Cunha G. Mangrich A. S. Romão L. P. C. (2019). Adsorption of aromatic compounds by biochar: influence of the type of tropical biomass precursor. Cellulose 26, 4291–4299. 10.1007/s10570-019-02394-0

[B8] Doltabadi M. Alidadi H. Davoudi M. (2016). Comparative study of cationic and anionic dye removal from aqueous solutions using sawdust-based adsorbent. Environ. Progress Sustain. Energy 35, 1078–1090. 10.1002/ep.12334

[B9] Gan Q. Allen S. J. Matthews R. (2004). Activation of waste MDF sawdust charcoal and its reactive dye adsorption characteristics. Waste Manag. 24, 841–848. 10.1016/j.wasman.2004.02.010 15381236

[B10] Han H. Rafiq M. K. Zhou T. Xu R. Mašek O. Li X. (2019). A critical review of clay-based composites with enhanced adsorption performance for metal and organic pollutants. J. Hazard. Mater. 369, 780–796. 10.1016/j.jhazmat.2019.02.003 30851518

[B11] Jang H. M. Kan E. (2019). Engineered biochar from agricultural waste for removal of tetracycline in water. Bioresour. Technol. 284, 437–447. 10.1016/j.biortech.2019.03.131 30981196

[B12] Jang H. M. Yoo S. Choi Y.-K. Park S. Kan E. (2018). Adsorption isotherm, kinetic modeling and mechanism of tetracycline on *Pinus taeda*-derived activated biochar. Bioresour. Technol. 259, 24–31. 10.1016/j.biortech.2018.03.013 29536870

[B13] Jeong J. Song W. Cooper W. J. Jung J. Greaves J. (2010). Degradation of tetracycline antibiotics: mechanisms and kinetic studies for advanced oxidation/reduction processes. Chemosphere 78, 533–540. 10.1016/j.chemosphere.2009.11.024 20022625

[B14] Li M.-f. Liu Y.-g. Liu S.-b. Zeng G.-m. Hu X. Tan X.. (2018). Performance of magnetic graphene oxide/diethylenetriaminepentaacetic acid nanocomposite for the tetracycline and ciprofloxacin adsorption in single and binary systems. J. Colloid Interface Sci. 521, 150–159. 10.1016/j.jcis.2018.03.003 29567603

[B15] Li X. Zhao Q. Feng X. Pan L. Wu Z. Wu X.. (2019). Pyridinic nitrogen-doped graphene nanoshells boost the catalytic efficiency of palladium nanoparticles for the N-Allylation reaction. Chemsuschem 12, 858–865. 10.1002/cssc.201802532 30600929

[B16] Lian F. Sun B. Song Z. Zhu L. Qi X. Xing B. (2014). Physicochemical properties of herb-residue biochar and its sorption to ionizable antibiotic sulfamethoxazole. Chem. Eng. J. 248, 128–134. 10.1016/j.cej.2014.03.021

[B17] Liu P. Liu W.-J. Jiang H. Chen J.-J. Li W.-W. Yu H.-Q. (2012). Modification of bio-char derived from fast pyrolysis of biomass and its application in removal of tetracycline from aqueous solution. Bioresour. Technol. 121, 235–240. 10.1016/j.biortech.2012.06.085 22858491

[B18] Liu S. Li M. Liu Y. Liu N. Tan X. Jiang L. (2019). Removal of 17β-estradiol from aqueous solution by graphene oxide supported activated magnetic biochar: adsorption behavior and mechanism. J. Taiwan Inst. Chem. Eng. 102, 330–339. 10.1016/j.jtice.2019.05.002

[B19] Liu W.-J. Zeng F.-X. Jiang H. Zhang X.-S. (2011). Preparation of high adsorption capacity bio-chars from waste biomass. Bioresour. Technol. 102, 8247–8252. 10.1016/j.biortech.2011.06.014 21724386

[B20] Liu X. Zhang W. Zhang Z. (2014). Preparation and characteristics of activated carbon from waste fiberboard and its use for adsorption of Cu(II). Mater. Lett. 116, 304–306. 10.1016/j.matlet.2013.11.062

[B21] Maleki A. Hajizadeh Z. Firouzi-Haji R. (2018). Eco-friendly functionalization of magnetic halloysite nanotube with SO_3_H for synthesis of dihydropyrimidinones. Microporous Mesoporous Mater. 259, 46–53. 10.1016/j.micromeso.2017.09.034

[B22] Marzbali M. H. Esmaieli M. Abolghasemi H. Marzbali M. H. (2016). Tetracycline adsorption by H_3_PO_4_-activated carbon produced from apricot nut shells: a batch study. Process Safety Environ. Protect. 102, 700–709. 10.1016/j.psep.2016.05.025

[B23] McKay G. Blair H. S. Gardner J. R. (1982). Adsorption of dyes on chitin. I. Equilibrium studies. J. Appl. Polym. Sci. 27, 3043–3057. 10.1002/app.1982.070270827

[B24] Nguyen V.-T. Nguyen T.-B. Chen C.-W. Hung C.-M. Vo T.-D.-H. Chang J.-H.. (2019). Influence of pyrolysis temperature on polycyclic aromatic hydrocarbons production and tetracycline adsorption behavior of biochar derived from spent coffee ground. Bioresour. Technol. 284, 197–203. 10.1016/j.biortech.2019.03.096 30939381

[B25] Pan R. Zou J. Li Y. Jin X. (2018). Hyperbranched polyethylenimine modified waste fiberboard activated carbon for enhanced adsorption of hexavalent chromium. J. Wood Chem. Technol. 38, 111–122. 10.1080/02773813.2017.1388820

[B26] Pan R. R. Fan F. L. Li Y. Jin X. J. (2016). Microwave regeneration of phenol-loaded activated carbons obtained from *Arundo dona*x and waste fiberboard. RSC Adv. 6, 32960–32966. 10.1039/C6RA01642A

[B27] Patra J. M. Panda S. S. Dhal N. K. (2017). Biochar as a low-cost adsorbent for heavy metal removal: a review. Int J Res Biosci. 6, 1–7.

[B28] Premarathna K. S. D. Rajapaksha A. U. Adassoriya N. Sarkar B. Sirimuthu N. M. S. Cooray A.. (2019). Clay-biochar composites for sorptive removal of tetracycline antibiotic in aqueous media. J. Environ. Manag. 238, 315–322. 10.1016/j.jenvman.2019.02.069 30852408

[B29] Regkouzas P. Diamadopoulos E. (2019). Adsorption of selected organic micro-pollutants on sewage sludge biochar. Chemosphere 224, 840–851. 10.1016/j.chemosphere.2019.02.165 30852464

[B30] Selmi T. Sanchez-Sanchez A. Gadonneix P. Jagiello J. Seffen M. Sammouda H. (2018). Tetracycline removal with activated carbons produced by hydrothermal carbonisation of *Agave americana* fibres and mimosa tannin. Indus. Crops Products 115, 146–157. 10.1016/j.indcrop.2018.02.005

[B31] Shaheen S. M. Niazi N. K. Hassan N. E. E. Bibi I. Wang H. Tsang D. (2019). Wood-based biochar for the removal of potentially toxic elements in water and wastewater: a critical review. Int. Mater. Rev. 64, 216–247. 10.1080/09506608.2018.1473096

[B32] Smyk B. Piotrowicz-Cieślak A. I. Grajek H. Rydzynski D. Margas M. Wasilewski J. (2019). Influence of light and Fe(III) ions on tetracycline degradation. Spectrochim. Acta A Mol. Biomol. Spectrosc. 216, 273–282. 10.1016/j.saa.2019.03.031 30904635

[B33] Wang H. Chu Y. Fang C. Huang F. Song Y. Xue X. (2017). Sorption of tetracycline on biochar derived from rice straw under different temperatures. PLoS ONE 12:e0182776. 10.1371/journal.pone.0182776 28792530 PMC5549735

[B34] Wang Y. Wang X. Li J. Li Y. Xia S. Zhao J. (2019). Coadsorption of tetracycline and copper(II) onto struvite loaded zeolite – an environmentally friendly product recovered from swine biogas slurry. Chem. Eng. J. 371, 366–377. 10.1016/j.cej.2019.04.058

[B35] Wu Y. Jin X.-J. Zhang J. (2013). Characteristics of nitrogen-enriched activated carbon prepared from waste medium density fiberboard by potassium hydroxide activation. J. Wood Sci. 58, 395–404. 10.1007/s10086-012-1312-4

[B36] Wu Y. Jin X.-J. Zhang M.-Y. Xu D. (2012). Phenol adsorption on nitrogen-enriched activated carbon from wood fiberboard waste. Wood Fiber Sci. 44, 220–226.

[B37] Wu Y. Zhang J. Jin X.-J. Gao J.-M. Zhao Q. (2014). Study of Cr(VI) adsorption onto nitrogen-enriched activated carbon from waste medium density fiberboard. Wood Sci. Technol. 48, 713–725. 10.1007/s00226-014-0632-5

[B38] Xiang Y. Xu Z. Wei Y. Zhou Y. Yang X. Yang Y.. (2019). Carbon-based materials as adsorbent for antibiotics removal: mechanisms and influencing factors. J. Environ. Manag. 237, 128–138. 10.1016/j.jenvman.2019.02.068 30784860

[B39] Zhan H. Zhuang X. Song Y. Liu J. Li S. Chang G. (2019). A review on evolution of nitrogen-containing species during selective pyrolysis of waste wood-based panels. Fuel 253, 1214–1228. 10.1016/j.fuel.2019.05.122

[B40] Zhang M.-y. Jin X.-j. Zhao Q. (2014). Preparation of N-doped activated carbons for electric double-layer capacitors from waste fiberboard by K_2_CO_3_ activation. New Carbon Mater. 29, 89–95. 10.1016/S1872-5805(14)60128-1

[B41] Zhang Y. Zhou J. Chen X. Wang L. Cai W. (2019). Coupling of heterogeneous advanced oxidation processes and photocatalysis in efficient degradation of tetracycline hydrochloride by Fe-based MOFs: synergistic effect and degradation pathway. Chem. Eng. J. 369, 745–757. 10.1016/j.cej.2019.03.108

