# Supplementary material for: Application of Biochar Derived From Pyrolysis of Waste Fiberboard on Tetracycline Adsorption in Aqueous Solution
Source: Front Chem. 2020 Feb 13;7:943. doi: 10.3389/fchem.2019.00943 (PMC7031499; doi:10.3389/fchem.2019.00943)
Supplement: Supplementary file 1 [file Data_Sheet_1.pdf]

Supplementary Material

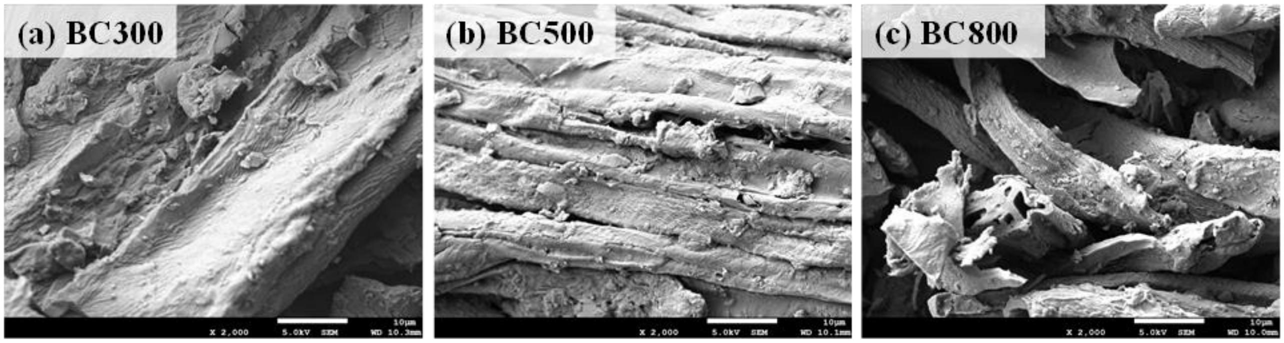

Figure S1. SEM images of a) BC300, b) BC500, and c) BC800. BCXXX stands for biochar prepared at XXX °C, XXX can be 300, 500 and 800.

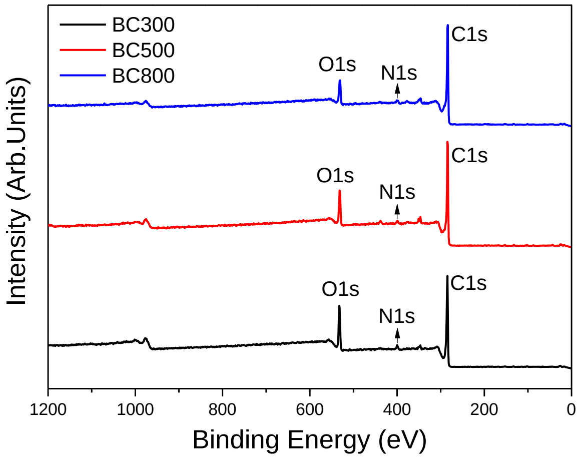

Figure S2. XPS wide scanspectra of BC300, BC500, and BC800. BCXXX stands for biochar prepared at XXX °C, XXX can be 300, 500 and 800.

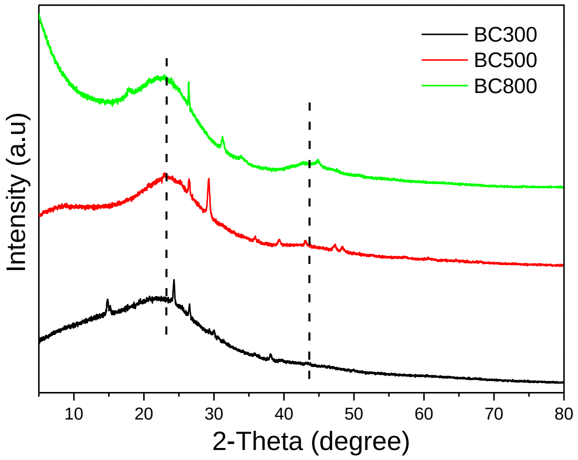

Figure S3. XRD patterns of BC300, BC500, and BC800. BCXXX stands for biochar prepared at XXX °C, XXX can be 300, 500 and 800.

Table S1. Comparison of maximum adsorption capacity of tetracycline by BC adsorbents derived from different biomass. BC stands for biochar.

BC adsorbents derived from different biomass	Q_m (mg/g)	Reference
Salix psammophila	2.71	(Zhu et al., 2014)
waste Auricularia auricular dregs	11.90	(Dai et al., 2020)
rice straw	13.85	(Wang et al., 2017)
Sewage sludge	13.92	(Yang et al., 2016)
swine manure	8.13	(Wang et al., 2018)
waste fiberboard	8.05	In this study

References

Dai, Y., Li, J., and Shan, D. (2020). Adsorption of tetracycline in aqueous solution by biochar derived from waste Auricularia auricula dregs. *Chemosphere* 238**,** 124432. doi: 10.1016/j.chemosphere.2019.124432.

Wang, H., Chu, Y., Fang, C., Huang, F., Song, Y., and Xue, X. (2017). Sorption of tetracycline on biochar derived from rice straw under different temperatures. *PLOS ONE* 12(8)**,** 1-14. doi: 10.1371/journal.pone.0182776.

Wang, H., Fang, C., Wang, Q., Chu, Y., Song, Y., Chen, Y., et al. (2018). Sorption of tetracycline on biochar derived from rice straw and swine manure. *RSC Advances* 8(29)**,** 16260-16268. doi: 10.1039/C8RA01454J.

Yang, X., Xu, G., Yu, H., and Zhang, Z. (2016). Preparation of ferric-activated sludge-based adsorbent from biological sludge for tetracycline removal. *Bioresource Technology* 211**,** 566-573. doi: 10.1016/j.biortech.2016.03.140.

Zhu, X., Liu, Y., Zhou, C., Luo, G., Zhang, S., and Chen, J. (2014). A novel porous carbon derived from hydrothermal carbon for efficient adsorption of tetracycline. *Carbon* 77**,** 627-636. doi: 10.1016/j.carbon.2014.05.067.
